# Metallothionein 3 Expression in Normal Skin and Malignant Skin Lesions

**DOI:** 10.1007/s12253-014-9805-7

**Published:** 2014-07-13

**Authors:** Bartosz Pula, Tadeusz Tazbierski, Aleksandra Zamirska, Bozena Werynska, Andrzej Bieniek, Jacek Szepietowski, Janusz Rys, Piotr Dziegiel, Marzena Podhorska-Okolow

**Affiliations:** 1 Department of Histology and Embryology, Medical University of Wroclaw, Chalubinskiego 6a, 50-368 Wroclaw, Poland; 2 Department and Clinic of Dermatology, Venereology and Allergology, Medical University of Wroclaw, Wroclaw, Poland; 3 Department of Pulmonology and Pulmonary Tumors, Medical University of Wroclaw, Wroclaw, Poland; 4 Department of Tumor Pathology, Maria Sklodowska-Curie Memorial Institute Oncology Center, Krakow, Poland; 5 Department of Physiotherapy, University School of Physical Education, Wroclaw, Poland

**Keywords:** Metallothionein-3, Skin cancer, Squamous cell carcinoma, Basal cell carcinoma

## Abstract

Metallothionein-3 (MT-3) has been shown to be expressed in several malignancies and to have an impact on patients’ survival in breast and urinary bladder cancer cases. However, its expression has not been determined in normal skin or in its malignant lesions. MT-3 expression was studied using immunohistochemistry in 17 cases of normal skin, 18 of actinic keratosis (AK), 39 of squamous cell carcinoma (SCC), and 23 of basal cell carcinoma (BCC). Low MT-3 expression was observed in normal skin epidermis with faint or no expression in the epidermis basal layer. Significantly higher MT-3 expression was noted in AK (*P* = 0.007) and SCC (*P* < 0.0001), as compared with normal skin epidermis. BCC cases were characterized by the lowest MT-3 expression of all the examined groups, which was significantly lower in comparison to normal skin epidermis, AK, and SCC (*P* = 0.009; *P* < 0.0001 and *P* < 0.0001, respectively). In conclusion, MT-3 may be involved in the development of SCC.

## Introduction

Nonmelanoma skin cancer (NMSC) is the most frequent form of neoplastic disease in Caucasian populations [1]. Since the 1960s, its overall incidence has increased by approximately 3–8 % per year [1, 2]. NMSCs are mainly represented by two histological tumor types: basal cell carcinoma (BCC) and squamous cell carcinoma (SCC) [1, 3]. Although BCC is seen up to five times more frequently in patients, it is considered to be an exclusively local malignant tumor due to the fact that metastases (to lymph nodes and other organs) are rarely observed [1, 4, 5]. However, SCC is considered a malignant skin tumor and is responsible for the majority of NMSC associated deaths [3]. Almost 80 % of all BCCs arise on sun-exposed skin, developing mainly on the head and neck regions [4, 5]. It has been shown that SCC may develop either from a precursor lesion, such as actinic keratosis (AK), or *de novo*. Although it is thought that a significant fraction of SCCs evolve from AK, the potential of malignant transformation cannot be predicted from clinical characteristics and is difficult to assess [3]. Unlike SCCs, BCCs do not have precursor lesions and instead appear *de novo* as a tumor which can usually be classified into one of two main groups: nodular BCCs are the most common, presenting as a small pearly papule with well-developed peripheral cells palisading. Superficial BCCs macroscopically manifest as scaly erythematous plaques and buds of atypical basal cells arising from the basal layer of epidermis [5, 6]. The abovementioned forms manifest low aggressiveness and may often be cured through local excision, however also histopathological BCC types of higher aggressiveness and frequent local recurrences are distinguished e.g. micronodular, morpheaform, infiltrative and metatypic types [5, 6].

Metallothioneins (MTs) are a family of small (6–7 kDa), intracellular, nonenzymatic family of proteins that are highly conserved among species [7]. MTs are characterized by high cysteine content in their polypeptide chain [8]. The high number of thiol (−SH) groups enables them to bind metals, such as Zn, Cd, Cu, and Hg, and to transfer Cu and Zn to the catalytic sites of various enzymes [9, 10]. MTs are encoded by a family of genes located on human chromosome 16q13, and four main groups of these proteins can be distinguished (MT-1, MT-2, MT-3, MT-4) [11]. Lines of evidence suggest that MT-1 and MT-2 play important roles in various cellular processes—in both normal and cancer cells—such as detoxification of heavy metals, homeostasis of zinc and copper ions, and protection of cell DNA against oxidative stress damage, proliferation, and apoptosis [7, 8, 10, 12–14]. Elevated MT-1/2 expression has been associated with poor patient prognosis in ovarian, renal, lung, and colorectal cancer, as well in soft tissue sarcomas [12, 15–20]. Although numerous studies have been performed on the role of MT-1/2 isoforms in various normal and pathological processes, little is known yet regarding the role of metallothionein-3 (MT-3) in cancer cells.

MT-3 was first identified in rat brain extracts in the course of Alzheimer’s disease and possesses an unique neuronal cell growth inhibitory property, not exerted by other members of the family, (such as MT-1/2) [21]. On account of this property, MT-3 was first described as a growth-inhibitory factor (GIF) [21, 22]. Its expression was initially reported to be very restricted to the nervous tissues, but further studies proved its wider distribution in the human body [22–28]. The results of recent research concerning the role of MT3 in neoplastic diseases remain ambiguous and are frequently inconsistent. It has been shown that, in human bladder, breast, and prostate cancer, the expression of MT-3 increases [22, 24, 27]. On the contrary, in esophageal squamous cell cancer and adenocarcinoma of the esophagus—as well as in gastric cancer—MT-3 expression decreases, as compared with normal tissue [23, 29, 30]. Although the expression of MT-1/2 has been intensively studied in normal and malignant skin lesions, no information concerning MT-3 expression is currently available [31, 32]. It has been shown that MT-1/2 expression increases significantly with the progression from normal skin to AK, with the highest expression noted for SCC [32]. Moreover, MT-1/2 has been shown to be positively correlated with Ki-67 antigen expression in AK, SCC, and BCC—confirming its role in regulation of cell proliferation [31, 32].

Taking into account that MT-1/2 may have an impact on carcinogenic processes in the skin, we analyzed the expression of MT-3 using immunohistochemical methods in cases of normal skin, AK, SCC, and BCC, with regard to the clinical and pathological characteristics of patients.

## Material and Methods

### Patients and Tissue Samples

The study was performed on tissue samples obtained from patients during the resection of skin lesions due to suspicion of clinical cancer in the Department and Clinic of Dermatology, Venereology, and Allergology, Medical University of Wroclaw. The study group consisted of 97 skin samples, of which 17 were diagnosed as normal skin, 18 as AK, 39 as SCC and, 23 as BCC (12 of the nodular type and 11 of the superficial type).

Resected tissue samples were fixed in 10 % buffered formalin and embedded in paraffin following prior dehydration. Six micrometer-thick sections were evaluated independently by two pathologists to verify the diagnosis and to assess the clinical and pathological characteristics of the analyzed SCC cases. The four-grade Broders scale classifies the differentiation and keratinization of tumor cells, and was employed here to determine the malignancy grade of the studied SCC tumors [33]. It is encoded as follows: G1 (>75 % keratinized cells), G2 (50–75 % keratinized cells), G3 (25–50 % keratinized cells), and G4 (<25 % keratinized cells). In addition, the degree of keratinization and the depth of inflammatory infiltrate were assessed. The mean patient age was 72.5 ± 13.2 (range 37 to 97) years. The clinical and pathological data of the SCC patients are summarized in Table 1.

**Table. 1:** Patient and tumor characteristics of the squamous cell carcinoma cases analyzed

Parameter	No. of cases (%)
Sex	
Male	22 (56.4 %)
Female	17 (44.6 %)
Differentiation	
Keratotic	31 (79.5 %)
Akeratotic	8 (20.5 %)
Broders scale	
G1	7 (17.9 %)
G2	10 (25.7 %)
G3	14 (35.9 %)
G4	8 (20.5 %)
Depth of inflammatory infiltration	
Epidermis	9 (23.1 %)
Subcutaneous tissue	30 (76.9 %)
Sun exposure	
Exposed	36 (92.3 %)
Occulted	3 (7.7 %)

### Immunohistochemistry (IHC)

For IHC reactions, four micrometer-thick sections were freshly cut and deparaffinized in xylene with subsequent rehydration in alcohol (absolute, 96 %, 70 %), with 5 min’ incubation each. Sections were boiled for 20 min in Target Retrieval Buffer (high pH for MT-3 and low pH for Ki-67 antigen) using a PT Link (Pre-Treatment Module for Tissue Specimens), in order to retrieve the studied antigens. Sections were then rinsed in TBS buffer with 0.05 % Tween. Then the EnVision™ FLEX Detection System was employed. EnVision™ FLEX Peroxidase-Blocking Reagent was used to block endogenous peroxidase, with 5 min’ incubation. Following rinsing in TBS with 0.05 % Tween, the primary antibodies were applied: anti-MT-3, a rabbit polyclonal antibody raised against GGEAAEAEAEKC peptide (1:200, Invitrogen, Carlsbad, California, USA) and anti-Ki-67 (ready to use, Dako Cytomation, Glostrup, Denmark). Following 20 min of incubation, the slides were rinsed in TBS with 0.05 % Tween, and the secondary antibody conjugated with horseradish peroxidase was used (EnVision™ FLEX/HRP), with incubation for 20 min. The MT-3 and Ki-67 reaction was visualized using freshly prepared substrate for horseradish peroxidase (diaminobenzidine, EnVision™ Working Solution), with incubation for 10 min. Additionally, all slides were counterstained using EnVision™ FLEX Hematoxylin and 5 min’ incubation. Eventually, after dehydration in graded ethanol concentrations (70 %, 96 %, absolute) and in xylene, all slides were closed with coverslips in SUB-X Mounting Medium in Coverslipper. All reagents and equipment, except for the MT-3 antibody, were purchased from Dako Cytomation.

### Evaluation of IHC Reactions

All IHC reactions were assessed using a BX41 light microscope (Olympus, Tokyo, Japan) independently by two pathologists, who were blinded to patient clinico-pathological data. In case of discrepancy between the pathologist, the staining was discussed until a consensus has been achieved. The expression intensity of MT-3 in the tumor cells was evaluated using the semi-quantitative 12-point immunoreactive score (IRS) of Remmele and Stegner, utilized previously in our studies concerning MT1/2 expression [31, 34]. The cytoplasmic MT3 IRS reaction score was calculated in each case, taking into account both the percentage of positive cells (0 points: no cells with positive reaction; 1 point: 1–10 % cells; 2 points: 11–50 %; 3 points: 51–80 %; 4 points: over 80 % cells with positive reaction) and the intensity of the reaction (0: no reaction; 1: low intensity of the reaction color; 2: moderate intensity of the reaction color; 3: intense color of the reaction). The final score represents the product of these two parameters and ranges from 0 to 12 points.

The expression of Ki-67 antigen in healthy skin and its lesions was assessed by the proportion of positive cells from all epidermal (normal skin and AK) or tumor cells (BCC and SCC) in whole tissue sections, and was coded as follows: 0 points for no reaction, 1 point for 1–10 %, 2 points for 11–25 %, 3 points for 26–50 %, and 4 points for > 50 % positive cells.

### Statistical Analysis

The results were subjected to statistical analysis using Prism 5.0 (GraphPad Software, La Jolla, CA, USA). The Shapiro-Wilk test was employed to test the distribution. Mann-Whitney’s *U*-test was used to assess the differences in MT-3 expression between the two groups. The correlation between the expression intensity of the selected markers was evaluated using the Spearman correlation test, or the Pearson test in the case of patient age analyses. The results were regarded as statistically significant for *P* < 0.05.

## Results

**Fig. 1:**
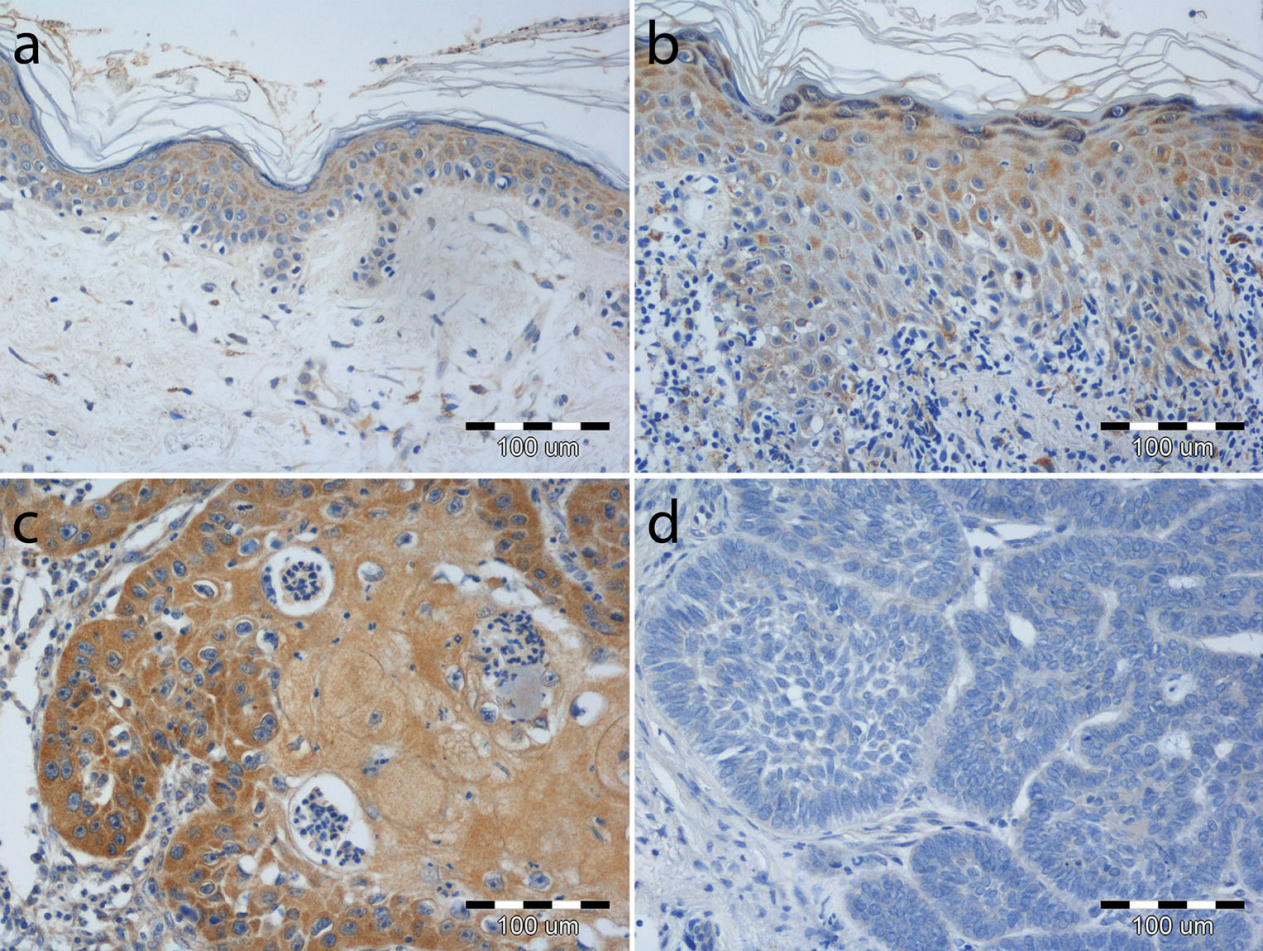
Cytoplasmic metallothionein-3 expression (**a**) in healthy skin epidermis, (**b**) in actinic keratosis, (**c**) in squamous cell carcinoma, and (**d**) in basal cell carcinoma. Magnification × 200

A cytoplasmic reaction in MT-3 immunostained slides was observed in 89 (91.8 %) of all analyzed cases. No staining was noted in the remaining 8 (8.2 %) cases (Fig. 1), one of which involved normal healthy skin and seven of which involved BCC. No nuclear MT-3 expression pattern could be seen in the keratinocytes of normal skin epidermis, AK, or in the tumor cells of BCC and SCC. MT-3 expression was mostly noted in the suprabasal layer of the epidermis in normal skin and AK lesions, with only scant faint expression noted in the basal layer (Fig. 1).

**Fig. 2:**
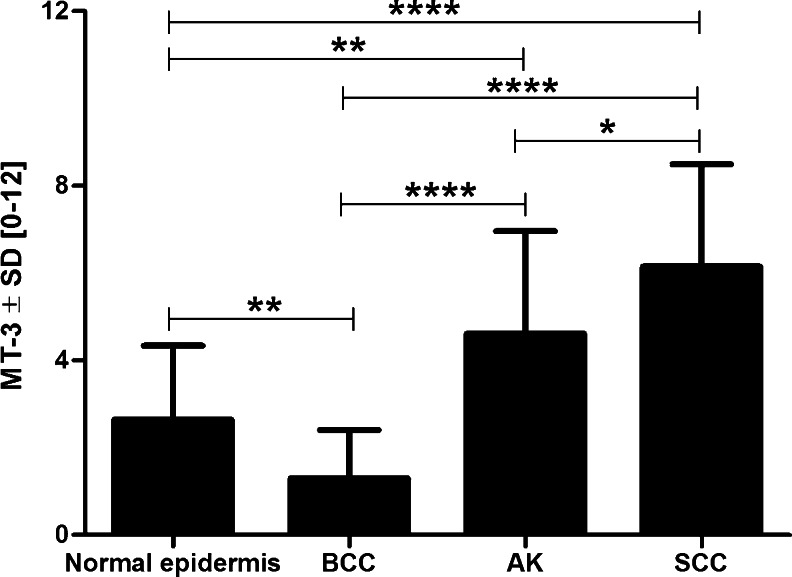
Differentiated cytoplasmic metallothionein-3 (MT-3) expression with regard to the type of analyzed sample. * *P* < 0.05; ** *P* < 0.005; *** *P* < 0.0005; **** *P* < 0.0001 (Mann–Whitney test); AK: actinic keratosis; BCC: basal cell carcinoma; SCC: squamous cell carcinoma

Low MT-3 expression level was observed in normal skin epidermis, scoring at the average of 2.65 (SD ± 1.69) on the IRS scale. A significantly higher MT-3 expression was noted in AK (IRS 4.61 ± 2.36; *P* = 0.007) and SCC (IRS 6.15 ± 2.35; *P* < 0.0001), as compared with normal skin epidermis. The BCC cases were characterized by the lowest intensity of MT-3 expression of all the examined groups (IRS 1.30 ± 1.11), being significantly lower in comparison to normal skin epidermis, AK, and SCC (*P* = 0.009; *P* < 0.0001 and *P* < 0.0001, respectively) (Fig. 2).

**Fig. 3:**
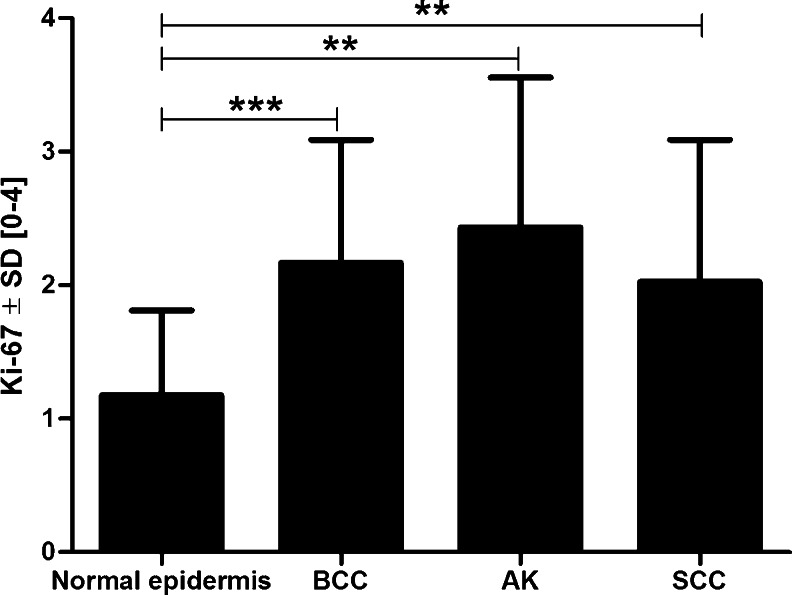
Nuclear Ki-67 antigen expression in regard to the type of analyzed sample. ** *P* < 0.005; *** *P* < 0.0005 (Mann–Whitney test); AK: actinic keratosis; BCC: basal cell carcinoma; SCC: squamous cell carcinoma

Nuclear expression of Ki-67 antigen was observed in all of the analyzed cases. Its expression was lowest in healthy skin epidermis (1.18 ± 0.64), and was significantly lower than in the cases of BCC (2.16 ± 0.92; *P* = 0.006), AK (2.17 ± 0.92; *P* = 0.002), and SCC (2.02 ± 1.06; *P* = 0.0062) (Fig. 3).

The statistical analysis of MT-3 expression in SCC cases did not reveal any significant differences in SCC as regarding patient age (Pearson correlation test), sex, depth of inflammatory infiltration, malignancy grade, differentiation (keratotic vs. akeratotic), or sun exposure (Mann–Whitney test, respectively). Similar results were observed for Ki-67 antigen expression. Moreover, no significant correlation was noted between MT-3 and Ki-67 antigen expression in normal skin and its lesions (BCC, AK, SCC; Spearman correlation test).

## Discussion

The MT-3 gene was first identified by Palmiter et al., and initial studies demonstrated that MT-3 expression was limited mainly to neuronal tissue [35, 36]. Nevertheless, recent research has revealed a broader physiological distribution of the molecule not only in normal tissue, but also in human prostate, esophagus, breast, and non-small-cell lung cancers [24, 25, 28, 37, 38]. Although MT-3 expression has been identified in cancer cells, its exact role in oncological diseases requires further clarification. Augmented MT-3 expression was observed in neoplastic cells of breast, prostate, bladder, and non-small-cell lung cancer [22, 24, 27, 38]. In contrast, in human esophageal cancers and gastric cancer, MT-3 expression has been seen to be frequently downregulated due to the increased DNA methylation in comparison with nonmalignant tissues of these organs [23, 29, 30].

In this study, we demonstrated for the first time MT-3 expression in normal skin keratinocytes. Additionally, its expression seems to be associated with the stratification of the epithelium, as cells in the basal layer are characterized by lower MT-3 expression levels (including zero expression) than in the case of the more differentiated cells of the suprabasal layers. Similar results have been obtained in regard to MT-4 [39]. In contrast, MT-1 and MT-2 probably exhibit different paths of expression and regulation, as less differentiated and undifferentiated cells from the basal layer demonstrate higher expression, as compared to suprabasal layers of keratinocytes [32].

Actinic keratosis is considered a premalignant skin lesion that can evolve to SCC [3, 32]. The observation of changes in the MT-3 expression intensity in normal skin epidermis, AK, and SCC point to the potential role of this protein in skin carcinogenesis. AK cases exhibit an intermediate MT-3 expression, which is higher in comparison to that noted in normal skin epidermis, but lower when compared to that of SCC cancer cells. Similar results regarding MT-1/2 were obtained by Zamirska et al., in whose work SCC cases were characterized by higher expression in AK, with the lowest expression in normal skin epidermis [32]. This observation may indicate the role of MT-3 in the development of SCC. Interestingly, we have also observed different intensities of its expression in cases of BCC and SCC, as significantly higher MT-3 expression was observed in the latter NMSC type. This observation is also consistent with the MT-3 expression pattern noted in healthy skin and AK lesions, in which only a scant, faint expression was visible in the basal cells. It is possible that MT-3 expression in BCC and basal cells may be the result of similar regulation processes based on DNA methylation, as has been observed in gastric and esophageal cancers [23, 30]. On the other hand, keratinocytes of the suprabasal layers are characterized by higher levels of MT-3 expression, which increases with the malignant potential of the lesion, the peak being achieved in SCC. These observations may be partially clarified by the regulation of zinc ion homeostasis in healthy and malignant skin lesions. Zinc ions have been shown to mediate numerous processes in skin biology, ranging from keratinocyte differentiation, through wound healing, to cancer development [40, 41]. Moreover, zinc deficiency was frequently present in patients with squamous cell carcinomas diagnosed in oral cavity, oropharynx and larynx [42]. MTs have been shown to regulate zinc ion homeostasis in response to stress, and therefore might serve as a key source of the exchangeable zinc ions needed for regulating transcription factor activity, and ultimately, diverse cellular response signals in SCC cancer cells [8, 43]. Although no studies have focused on revealing the relationship of MT-3 with metal ion homeostasis, this path in the carcinogenesis of the skin remains to be clarified. In this study we aimed at defining the role of MT-3 in the regulation of cellular proliferation, which was assessed in this study by examining the expression intensity of the Ki-67 antigen. However, no correlation between those two markers could be disclosed, indicating that MT-3 may probably impact other cellular process than proliferation in skin lesions. Recently, MT-3 was shown to be involved in the regulation of lysosomal function and autophagy in neurons and astrocytes, and may also potentially influence this processes during skin carcinogenesis [44, 45]. To our knowledge, the exact role of MT-3 expression in regard to autophagic processes in cancer cells has not yet been investigated. MT-3 was also shown to protect neurons from hypoxia or DNA-induced damage via increased activity of NF-κB and protein kinase B (Akt) signaling pathway [46]. This may also be one of potential mechanism of actions of MT-3, as it was shown that Akt is overexpressed in SCC [47].

In our study, we did not observe any significant relationship between MT-3 expression in SCC cancer cells and any clinical and pathological parameters that might point to the role of MT-3 in SCC progression and prognosis. Our results are comparable to those obtained during the examination of esophageal and gastric cancers, where MT-3 expression had no impact on patients’ clinical or pathological data or on patient survival [23, 29, 30]. However, in the study of Sens et al., MT-3 expression was up-regulated in human bladder cancer tumor cells, and was positively correlated with the tumor malignancy grade [27]. In addition, in breast cancer, MT-3 expression is considered to be a marker of poor prognosis [22, 37]. Although, we did not notice any correlations between MT-3 expression intensity in cancer cells and SCC patients clinico-pathological data, the prognostic impact of MT-3 expression remains to be yet determined. In the current study we had no information concerning the survival time of SCC patients, therefore we could not definitely define the potential utility of MT-3 as a new prognostic marker of this malignancy.

In summary, we have shown for the first time that MT-3 may be potentially involved in the development of SCC. Moreover, the varying expression levels of MT-3 observed by us in SCC and BCC confirm the differing biology of both these malignant skin lesions.
